# The pregnancy experience of Korean mothers with a prenatal fetal diagnosis of congenital heart disease

**DOI:** 10.1186/s12884-018-2117-2

**Published:** 2018-12-03

**Authors:** Yu-Mi Im, Tae-Jin Yun, Il-Young Yoo, Sanghee Kim, Juhye Jin, Sue Kim

**Affiliations:** 1 Department of Nursing, Seoul Women’s College of Nursing, Seoul, South Korea; 2 Division of Pediatric Cardiac Surgery, Asan Medical Center, University of Ulsan College of Medicine, Seoul, South Korea; 3 College of Nursing, Yonsei University, Seoul, South Korea; 4 College of Nursing, Mo-Im Kim Nursing Research Institute, Yonsei University, 50 Yonsei-ro, Seodaemun-gu, Seoul, 03722 Korea; 5 Department of Nursing, College of Health and Life Science, Korea National University of Transportation, Jeungpyeong-gun, South Korea

**Keywords:** Congenital heart disease, Prenatal diagnosis, Pregnancy, Experience, Grounded theory

## Abstract

**Background:**

Prenatal diagnosis of fetal congenital heart disease (CHD) is becoming widely available but there is a lack of understanding on such expectant mothers’ experiences during pregnancy. This was the first study to investigate the pregnancy experience of Korean mothers with a prenatal fetal diagnosis of CHD.

**Methods:**

In-depth interviews were conducted with 12 mothers regarding their child’s prenatal diagnosis of CHD and the adaptive processes during pregnancy. The data were transcribed and analyzed according to the grounded theory framework.

**Results:**

When the diagnosis of fetal CHD was suspected, mothers desperately sought accurate information regarding CHD while hoping in vain for a misdiagnosis. When the definitive diagnosis was made, most pregnant women experienced psychological trauma and pain, framed in the stigma and burden of having an imperfect child. Provision of accurate health advice and emotional support by a multidisciplinary counseling team was crucial at this phase, forming recognition that CHD could be treated. When fetal movements were felt, mothers came to acknowledge the fetus as an independent being, and made their best efforts to protect the fetus from harmful external influences using traditional *TaeKyo* mindset and practices, which in turn, were helpful in restructuring the meaning of the pregnancy.

**Conclusions:**

Mothers went through a dynamic process of adapting to the unexpected diagnosis of CHD, which was closely linked to being able to believe that their child could be treated. Early counseling with precise information on CHD, continuous provision of clear explanations on prognosis, sufficient emotional support, and well-designed prenatal education programs are the keys to an optimal outcome.

## Background

The incidence of congenital heart disease (CHD) is estimated as 8.8 in 1000 liveborn infants [1]. Congenital malformations are one of the leading causes of infant death in the United States and other developed nations, and critical CHD is responsible for more deaths than is any other type of malformation [2].

Among prenatal examinations performed during pregnancy, fetal echocardiography can be performed at first trimester [3, 4]. The cardiac screening examination, however, is performed optimally between 18 and 22 weeks’ gestational age and many anatomical structures can still be visualized satisfactorily beyond 22 weeks [5]. If fetal CHD is suspected, pregnant women are transferred to a tertiary medical institution that can provide an accurate prenatal diagnosis, specialized birth plans, neonatal intensive care, and emergency cardiac surgeries to increase the viability of the fetus [6–8].

Prenatal diagnosis of congenital anomaly creates an idiosyncratic situation. For newborns who do not have additional risk factors and their families pursued treatment, prenatal diagnosis reduced the risk of death with planned cardiac surgery [9]. But pregnant women who become aware of fetal CHD experience deep grief, anger, loss of affection for the fetus, and worry owing to the lack of information regarding the disease as well as its uncertain future [10]. A substantial proportion of mothers have been reported to exhibit evidence for traumatic stress, with nearly 40% exceeding clinical cutoff values for posttraumatic stress disorder [11]. Moreover, the parents may blame each other or feel guilty, thus leading to discord between them [12].

During this difficult phase, pregnant women consult with a variety of healthcare professionals to obtain information regarding the disease. Counseling provided by healthcare professionals is remarkably influential in the decision-making process [13, 14] and the particular role of nurses, such as empathizing with the parents’ emotions, coordinating with other healthcare professionals on behalf of parents, and supporting the parents’ decisions has also been reported [15]. Fully understanding the experience of women with fetal CHD, the disease, and its treatment trajectory is required of health professionals to provide high-quality healthcare. However, there have been no such studies in Korea, despite increasing numbers of CDH diagnosed through prenatal screening. Thus, the purpose of this study was to explore the overall pregnancy experience of women starting from the time when they were told of definite diagnosis of fetal CHD, up to when they gave birth, exploring the context of accepting and deciding to continue with the pregnancy.

## Methods

This study used grounded theory to explore and understand the pregnancy experience of mothers who had been told that their child has CHD. Since the primary purpose of grounded theory is to explore the dominant social processes that exist within human interactions [16], this method was considered most appropriate for our study. We attempted to assess how the mothers, as human beings, developed the meanings of each event and communicated with their inner selves in a variety of situations they encountered over the course of their pregnancy. These meanings were constructed through social experience and self-reflection, and by assigning such meanings to events the mothers could determine their responses [16].

### Participants

Mothers with a prenatal fetal diagnosis of CHD diagnosed by a specialist, who were willing to talk about their pregnancy experience were invited to participate in this study. Mothers were recruited through purposeful and theoretical sampling [16]. Exclusion criteria were infants with conditions associated with genetic disorders, such as Down’s syndrome, and if there were other congenital defects, such as cleft lip and palate.

The interval between birth and participating in the interviews did not exceed 6 months, in order to minimize the influence of parenting after birth on the mothers’ recollection of the pregnancy experience. The timing of interviews was carefully selected to avoid adding stress/anxiety to participating mothers, which could influence their recall. The issue of time frame (intervals ranging from 1 to 6 months), from birth to time of interview, depended on when the participant wished to be interviewed. Many interviews (*n* = 6) were done in the out-patient department, following the infant’s surgery, upon individual contact and setting a time. The remaining half (*n* = 6) were done in the hospital when the infant’s condition was stable (e.g., the day before expected discharge). Of the total of 12 mothers who participated in in-depth interviews, five mothers participated within 3 months and seven mothers between 4 and 6 months postpartum. The gestational age of the fetus at prenatal screening differed between mothers, ranging from the 21st to 33rd week of pregnancy.

### Data collection

In-depth interviews were undertaken from February to November 2013, following approval by the institutional review board of the tertiary level hospital from which mothers were recruited. Mothers provided written consent regarding voluntary participation and publishing the data in anonymous form. To collect enough vivid descriptions regarding the pregnancy experience, the main question was “Let’s recall the day when you were told that your baby had congenital heart defects. How did you feel about that and what was your reaction?” Most interviews were conducted in locations where participants felt comfortable, such as in their homes, at a counseling office in the hospital, or at the bedside of the infants following cardiac surgery. The researcher met the participants in casual attire rather than a hospital uniform to facilitate a natural, comfortable ambiance. Each mother participated in one to four interviews, with most participating two to three times. The first interview lasted for 1–2 h on a face-to-face basis, and additional interviews lasted for 30–60 min, either in person or by telephone. The researcher also took field notes and compiled analytical memos following the interviews, as a means of clarifying salient issues and preparing for the next interview.

### Data analysis

The data were analyzed based on the methodology proposed by Strauss and Corbin following open coding, axial coding, and selective coding, and we proceeded simultaneously with the process of data collection, abiding by the constant comparative method at the stage of coding mentioned above [17]. This iterative process made it possible to compare the concepts generated during the process of data collection with the pre-existing concepts and to discover their differences and similarities.

The interviews were recorded with consent having been obtained from the participants. Recorded interviews were transcribed by an assistant, and the researcher double-checked the recording to validate or modify the transcripts. All interviews were conducted and transcribed in Korean, and analysis was carried out in Korean, in order to recognize subtle nuances and cultural contexts. The themes that emerged from the study were translated into English at the final stage, and verified by the research team for accuracy and relevance.

## Results

### General characteristics of the participants

The general characteristics of the 12 mothers who participated in this study are presented in Table 1. The median age of the participants was 31.5 years. Half were stay-at-home mothers, and the remainder had professional occupations such as bankers, teachers or others. For most participants, this was their first pregnancy. The gestational age when a fetal diagnosis of CHD was made ranged from 21 to 33 weeks, with the first counseling being provided by the hospital’s counseling team between 22 and 36 weeks.

**Table 1:** General characteristics of the participants

Participant	Age group	GA at diagnosis (weeks)	GA at fetal counseling (weeks)	Timing of first interview (months)	Employment status	Birth order	Diagnosis
1	30s	23	23	6	Yes	1st	TGA with VSD, PS
2	20s	25	32	3	No	1st	FSV (AVSD, DORV, PS, TAPVR, Rt isomerism)
3	30s	23	26	6	Yes	1st	Truncus arteriosus
4	30s	25	26	5	Yes	1st	FSV (TA)
5	40s	27	27	3	Yes	2nd	TGA with VSD, PDA
6	30s	23	23	6	No	1st	FSV (Rt. Isomerism, DORV, CAVSD, PS, TAPVR
7	30s	34	37	3	No	1st	HLHS
8	30s	22	22	6	No	1st	DORV, VSD, CoA
9	20s	21	25	1	No	1st	FSV (DORV, DIRV, PS, ASD, rudimentary LV)
10	20s	25	26	4	No	1st	TOF
11	30s	23	32	1	Yes	2nd	PA with VSD, MAPCA
12	30s	22	27	4	Yes	1st	DORV, ASD

*GA* gestational age, *TGA* transposition of the great arteries, *VSD* ventricular septal defect, *PS* pulmonary stenosis, *FSV* functional single ventricle, *AVSD* atrioventricular septal defect, *DORV* double outlet right ventricle, *TAPVR* total anomalous pulmonary venous return, *Rt* right, *TA* tricuspid atresia, *PDA* patent ductus arteriosus, *HLHS* hypoplastic left heart syndrome, *CoA* coarctation of the aorta, *DIRV* double inlet right ventricle, *ASD* atrial septal defect, *LV* left ventricle, *TOF* tetralogy of Fallot, *PA* pulmonary atresia, *MAPCA* major aorto-pulmonary collateral artery

### Progression of pregnancy in mothers with prenatal diagnosis of CHD

**Fig. 1:**
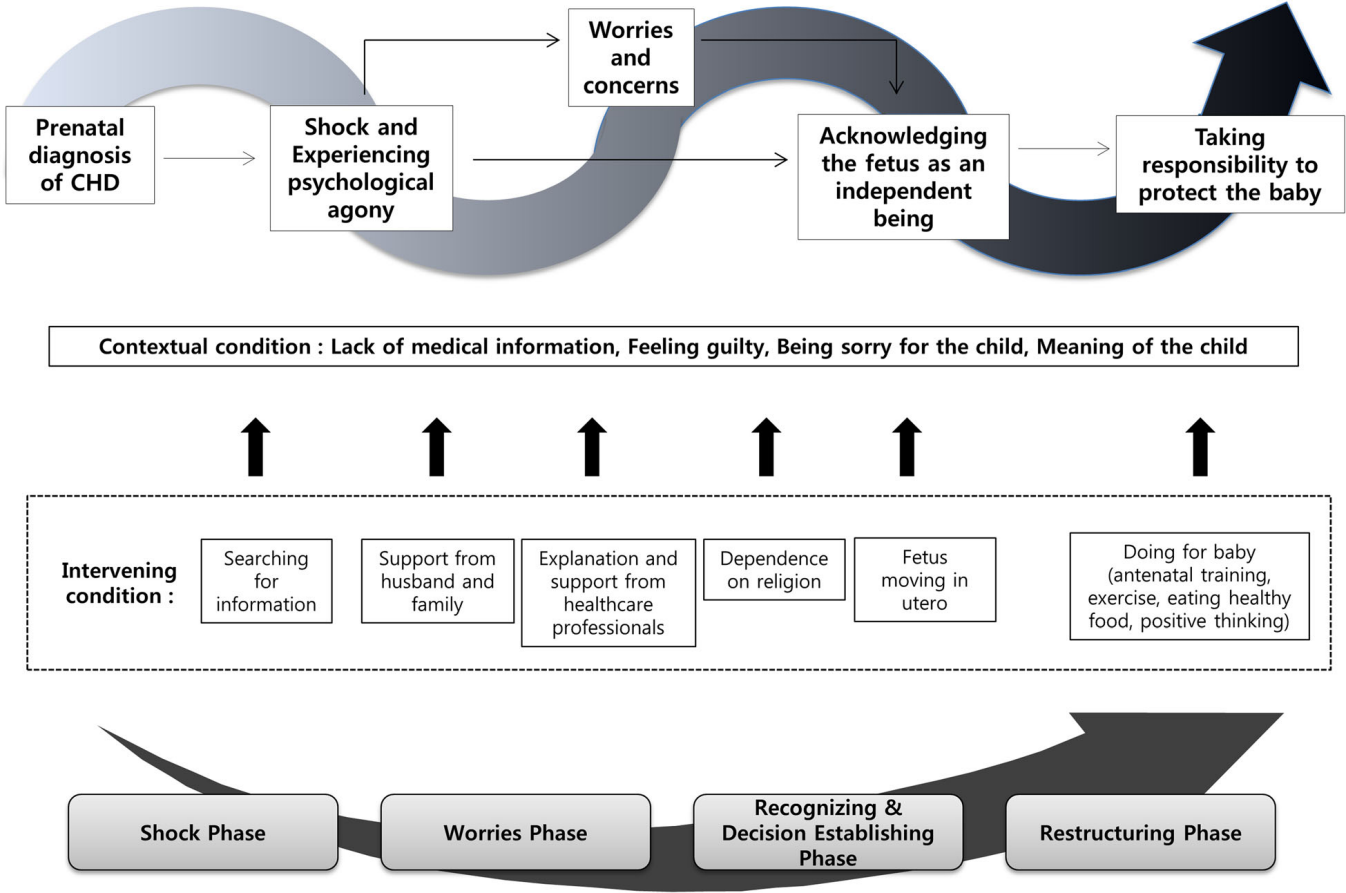
Adaptive process of Korean mothers with a prenatal fetal diagnosis of congenital heart disease

The chronological process following initial suspicion and confirmation of fetal CHD progressed along four phases, depicted by wavy arrows that reflect mothers’ fluctuating state: “Shock and pain from frustration and despair,” “Phase of worries,” “Recognition of the baby as a living human being,” and “Restructuring and endeavoring to protect the baby” (Fig. 1). Mothers initially experienced psychological trauma upon the prenatal diagnosis of CHD of their child, suffered from inner conflicts between maintaining and terminating the pregnancy, became determined to maintain the pregnancy after becoming aware of fetal movement, and finally restructured their pregnancy experience of conceiving a fetus with cardiac anomalies. However, not every study participant underwent all of these phases consecutively, and some of them bypassed the first or second phases.

#### Phase of shock and pain

Because mothers had never imagined that their child would have any problem, they were shocked when they first heard about CHD. Mothers asked themselves what they had done wrong before their pregnancy and why this was happening to them. They were unable to determine what their next step should be and were greatly concerned about the fact that they knew nothing anything about the defects. Some did not even want to accept this baffling situation.

*Why did this happen to me? My husband and I have never seen people around us who had babies with congenital heart defects. We were really sorry that God gave us a critically ill baby.*

*I felt as if someone was hitting my head time and again, telling me that I would not be able to bear it. Actually, I thought to myself again and again that I could not accept this. Giving birth to a baby with an abnormal heart would never be a thing I could handle … never ever!*

In their search for as much information as possible about the cardiac anomalies, they often experienced difficulty in making sense of the information found online. Thus, the most prominent feelings reported by the pregnant mothers after the confirmation of fetal CHD were uncertainty, difficulty in accepting the diagnosis, psychological stress, depression, and loneliness. Two influential modes to help them overcome such difficulties at this phase were acquisition of precise information about the heart anomaly, and support from the husband and family members.

#### Phase of concern and worries

Although some mothers did not go through this phase, seven out of 12 participants entered the second stage of inner conflict immediately after being shocked by the recognition of fetal heart problems. They began to agonize over whether or not they should maintain the pregnancy, as it conflicted with their cultural sense of duty to produce a healthy child. Termination of the pregnancy was occasionally proposed by the husband or other family members, framed in the stigma and burden of raising an imperfect child, which added to their distress and worries.

*While I saw my baby as merely being sick, my mom thought the baby was completely ‘abnormal,’ like an alien. I could understand my mom because she is an old woman, not knowing about state-of-the-art medical technology. I thought my sister would see this matter differently. However, I was freaked out when my sister cautiously asked me whether I might consider therapeutic abortion. She said ‘You’ll be able to be pregnant easily next time, so why do you want to deliver an imperfect baby?’*

*Considering the baby, I felt I should go ahead with maintaining the pregnancy. Considering the rest of my life, however, I shouldn’t. My thoughts were wandering from this side to that side like a pendulum. Thinking of my husband was the most painful part of my dilemma. When we got married, I promised I’d bear him a pretty and healthy baby … I could have quietly given up the baby without him knowing at all. Actually, I knew several hospitals which could have done the job for me, but…*

Some mothers immediately showed a determined attitude toward maintaining their pregnancy. For instance, women who became pregnant after failing several artificial fertilization procedures or having experienced miscarriages said they did not for a moment consider giving up the baby. Another participant stated that she did not consider an abortion because the fetus was a blessed gift from God, no matter how sick. Being able to have strong confidence in postnatal medical care and seeking assurance in religious beliefs were noted as influential ways to overcome the difficulties in this phase.

#### Recognition of the child as a living human being

Korean culture typically counts the baby’s time spent in utero and the baby becomes 1 years-old at birth [18]. Such assumption of the fetus’s being is tied to the practice of encouraging early prenatal interactions with the fetus, referred to as *TaeKyo*, a traditional concept thought to contribute to fetal development by focusing the pregnant mother’s mind and behaviors throughout the pregnancy [19]. Although *TaeKyo* was not a focus in the initial phases of shock and worry, feeling fetal movements (quickening) brought the actual recognition of the fetus as a living human being. This propelled the mothers to more easily accept the baby and this phase was characterized by a shift to determined attitude to maintain the pregnancy. The most important intervention to overcome difficulties at this stage was the feeling of being connected with the baby through fetal movements.

*It was a marvelous experience. I suddenly realized that something alive was in me. When I saw the ultrasound image of the baby’s face, this realization became even more vivid and made me extremely happy.*

*The baby was most actively moving when I was driving. I used to talk to her ‘Stop it!’ If she was not moving, then I tapped my belly and talked to her again, ‘Hey, what are you doing? Are you sleeping?’ As a matter of fact, I became nervous when she was not moving, thinking that something might go wrong. This ‘hide-and-seek’ game continued till the day she was born. I felt happy when she was kicking my belly. I felt as if she was sending me a signal that ‘Mommy, I’m here, and I will be OK later on after I’m born!*

#### Restructuring the pregnancy experience

At this phase mothers began to actively engage in a *TaeKyo* mindset, i.e., maintaining a peaceful, optimistic, and affirmative attitude, and began to ease away from the difficulties they had been through and form a different idea regarding the meaning of conceiving a child with CHD. In this phase mothers actively took on responsibility to protect the fetus via specific *TaeKyo* practices, such as eating nutritious foods and working out regularly in hopes of optimizing the postnatal condition of their child. As such, actively enacting *TaeKyo* mindset and practices was a way to restructure and solidify the meaning of their pregnancy and overcome prior difficulties.

*Yoga was one of the things I started to do after I decided to hang on to my baby. They taught me how to do abdominal breathing, and I felt as if my baby’s heart and lung function improved as I breathed using my abdominal muscles. I don’t know the scientific basis of this, but I just wanted to help my baby overcome the difficult surgeries after birth using his strong heart and lungs.*

*On prenatal counseling, I learned that the birth weight needs to be good enough for my baby to safely undergo the planned surgical procedures. The counselor told me I should eat meat, fruits, vegetables, and other nutritious foods as much as I could. I didn’t like to eat meat before pregnancy, and I didn’t even get to eat any meat after conception due to morning sickness. However, I began to eat meat every day, hoping that the birth weight would be greater than 3 kilograms. It was the only thing I could actually do for my baby.*

## Discussion

This was the first study to explore the experience of Korean women with a prenatal fetal diagnosis of CHD, starting from time of confirmed diagnosis to their adaptive process over time. Mothers within 6 months of delivery were invited to participate as we felt that including longer postnatal periods could incur greater recollection bias. The primary author’s clinical experience with such mothers provided assurance that this time frame (3–6 months) allows for basic adjustment of the mother-infant while still being a valid time point for thinking back on their pregnancy. The pregnancy experiences of mothers is this study can be understood as the process of transition, from initial shock and agony to endeavoring to protect their child. It was obvious that they initially had a difficult time and experienced pain and shock. However, as the mothers finally acknowledged the fetus as an independent being, they began to do their utmost to protect their fetus from any harmful influences that might prompt the termination of the pregnancy.

When the mothers were told that their child had CHD, the majority were initially in shock, hoped in vain for a misdiagnosis, and attempted to gain information regarding the CHD. These findings were consistent with previous reports from Western countries that pregnant women who discover congenital deformities in their unborn child experience shock regardless of the severity or curability of the malformation [20, 21]. In a study investigating the experience of expectant mothers discovering fetal congenital deformities, it was difficult even for mothers who were professional nurses to accept the unexpected diagnosis [10].

Our study confirmed the strong influence of spousal and family members’ attitude early on at diagnosis. We found this especially in relation to culturally engrained beliefs about gendered duties and social norms about the potential responsibility of the mother. This is parallel to studies in Asia that have noted that if a baby is born prematurely or with a low birth-weight, the mother may even be blamed by family or relatives for not observing the cultural practices appropriately, thus causing physical harm to the fetus [22]. Blame and stigma in Confucian-based cultures such as Korea should be considered and healthcare professionals can actively involve husbands and family members to understand CHD first-hand, and engage them to support the expectant mother.

Fetal movements, which can strengthen the bond between mother and child [23], were especially influential in facilitating the process of acceptance, and can be used by healthcare professionals as a point of reference when interacting with mothers of fetal CHD. Cultural practices of *TaeKyo* were tangible ways to solidify restructuring of the meaning of the pregnancy, likely related to its self-regulatory nature [24]. In addition to encouraging psychological and emotional preparations for becoming a mother, *TaeKyo* also has positive influence on maternal identity after childbirth [19]. *TaeKyo* reflects the responsibility of not only the expectant mother but also of the family and surrounding community [18], and can be a way to strengthen maternal-fetal attachment and self-efficacy in dealing with high risk pregnancy situations [24]. Healthcare professionals working with Korean mothers can encourage the cultural code of *TaeKyo* not only in its behavioral aspect aiming to maximize healthy fetal development, but also as a way to encourage development of a new view of the pregnancy as well as maternal identity.

In the initial phase of learning of the diagnosis, quick referral to a specialist and timely counseling is most important, supplemented by regular contacts with healthcare professionals. In the early phase, healthcare professionals can actively provide mothers and family members with accurate knowledge of the pathophysiology of CHD to enable informed decision-making regarding maintaining the pregnancy. Seeking information is a common way to cope with a stressful event, and previous studies have consistently described the significance of as much information as possible at the point of diagnosis [14, 25, 26]. When having confidence that CHD is curable, expectant mothers were able to accept the diagnosis and began to do whatever they could to protect the “baby while in my body”. In line with reports that mothers with fetal congenital anomaly needed more information throughout their pregnancy than their counterparts with a healthy pregnancy [20], during the course of pregnancy, our participants continuously searched for useful information and also sought information regarding the postnatal treatment process. However, the sources were often cases posted on online communities and it is imperative that maternal and neonatal professionals be a direct source of information through regular counseling. Emergency phone numbers may also be provided to allow mothers consistent access to information and support during pregnancy and after birth. The rapport established during pregnancy can continue to grow after childbirth and can have further positive impact on the continual process of their infant’s treatments.

Nearly all the infants in this study had serious conditions of CHD. Yet as the purpose of this study was to explore the general pregnancy experience of mothers with prenatal diagnosis of fetal CHD, we did not analyze whether disease severity might have exerted an influence. We found that most mothers interviewed did not seem to fully grasp the implications of severity, but struggled with the diagnosis itself, which essentially prompted this exploratory study. Future studies could further determine whether the pregnancy process is different according to the severity of heart disease or whether it may differ depending on timing of diagnosis or counseling period.

## Conclusions

Childbirth is considered as one of the most important life events not only for a mother, but a family as a whole. This research found that pregnant Korean women who learned that their fetus had CHD through prenatal screening experienced pain and shock, worry, recognition of the fetus as a human being, and, finally, restructuring of the pregnancy experience. Interventions by the healthcare professionals in this field need to be specific for each phase of transition. As better understanding is gained of the specific psychological transition undergone by these pregnant women, more appropriate and timely intervention may be enabled. At the initial point of encounter, healthcare providers can seek establishing a secure rapport with the mother, her spouse, and the immediate family. Clinical implications for healthcare providers also include the provision of clear information that is built up and repeated throughout the pregnancy so that expectant mothers can have factual and well-grounded understanding that CHD is treatable, thus avoiding an overly pessimistic view of the future. While the findings of this study may be more applicable to first time pregnancies, creating well-designed education programs and/or support groups for these mothers may be helpful measures that provide continuity and support. As spirituality and religiosity was an important aspect for this sample of Korean mothers, modes of spiritual expression may also be encouraged, and support from their faith community may provide additional benefit for these women.
